# Editorial: Gender Differentials in Times of COVID-19

**DOI:** 10.3389/fpsyg.2022.901087

**Published:** 2022-05-23

**Authors:** Michèle Belot, Stephan Müller, Holger A. Rau, Christiane Schwieren

**Affiliations:** ^1^Cornell University, Ithaca, NY, United States; ^2^University of Göttingen, Göttingen, Germany; ^3^Heidelberg University, Heidelberg, Germany

**Keywords:** COVID-19, gender differences, preferences, health protection, educational/career choices, gender inequality

## Why Gender Differentials Matter During The Pandemic

The COVID-19 pandemic constitutes a large exogenous shock for economies and societies worldwide. The crisis affected policymakers, firms, and households to similar extents. Because of the health threat, the primary goal was to fight the dissemination of the virus. As a consequence, the society experienced drastic changes due to policy measures such as social-distancing rules, lockdowns, school and university closures, and restricted access to public places. Furthermore, vivid discussions on legal vaccination requirements drove a division of society, stimulated conspiracy theories (e.g., Bierwiaczonek et al., 2020; Pummerer et al., 2022), and political polarization (e.g., Hart et al., 2020; Kerr et al., 2021). Compliance to these policy measures is important to preserve a healthy society with functioning labor markets, access to human capital in schools and universities, which guarantees growth (Keser and Rau, 2022). Social sciences may provide valuable insights, as the success of these measures depends on individual behavior. In this respect, people's preferences (Campos-Mercade et al., 2021; Müller and Rau, 2021), their perception of the crisis, and their socioeconomics are important factors that influence behavior.

Social psychology and behavioral economics emphasize evidence of gender differences in preferences (Croson and Gneezy, 2009; Meyers-Levy and Loken, 2015) that may play a crucial role for the observed outcomes during the Corona crisis. Women are consistently found to be more risk averse (Charness and Gneezy, 2012), less competitive (Niederle and Vesterlund, 2007), and more prosocial (Eckel and Grossman, 1998; Branas-Garza et al., 2018) and empathic (Mesch et al., 2011) than men. Transferred to the pandemic, which constitutes a risky situation where egoistic behavior induces negative externalities on others, it follows that gender differences in compliance could exist. In line with this argument, it is found that women are more likely to wear a face mask (Capraro and Barcelo, 2020) and to agree and comply with restraining public policy measures (Galasso et al., 2020) than men. Focusing on the labor market during the pandemic recession, the decline in employment is more pronounced for women (Albanesi and Kim, 2021), as they spend more time at home for child care, which is in line with less competitive behavior—but also, and this should not be forgotten, with structural and normative differences.

The papers mentioned above are examples demonstrating how gender differences in behavior may impact the outcomes during the pandemic, regarding compliance and behavior on labor markets. This Research Topic extends this evidence, contributing to the literature by analyzing gender differentials and their consequences in times of the COVID-19 crisis.

## This Research Topic

This Research Topic encompasses 10 articles that apply data from survey studies and online experiments to answer their research questions on gender differentials in the COVID-19 pandemic. The content ranges from contributions that analyze gender differences in the perception of remote teaching (Korlat et al.), in the perception of risk and the stability of risk preferences (Alsharawy et al.; Zhang and Palma), in partisanship Antinyan et al.), up to psychological aspects, such as symptoms of depression (Abreu et al.), and stress of expectant and postpartum parents (Tavares et al.). Further studies analyze the impacts of lockdowns on family life (Biroli et al.), and on potentially addictive behaviors (Attanasi et al.), as well as gender and wealth differences with respect to the allocation of scarce medical resources (Michailidou). Finally, Morgan et al. present a literature review on the different ways how all genders are affected by COVID-19.

Findings reveal that Austrian school girls report a higher perceived teacher support than boys (Korlat et al.). Regarding the perception of risk during the pandemic, Alsharawy et al. find in a US data set that women report greater fear and more negative expectations on health-related consequences of COVID-19 than men, while Zhang and Palma find in an MTurk study that general risk preferences of women and men and their difference seem to be stable during the COVID-19 crisis. Antinyan et al. show in their US survey experiment that exposing subjects to alternatives narratives on the causes of the pandemic increases the partisanship gender gap, since women become more liberal. Several papers report gender differences in the effects of countermeasures against the pandemic, and specifically lockdowns: Abreu et al. present evidence of German cross sectional data (“Live with Corona” survey), which suggests that COVID-19 and its countermeasures are associated with a stronger increase in aggression for men than for women. Tavares et al. find in a Portuguese online survey with expectant parents that men under lockdowns report higher levels of stress than those who were not exposed to lockdowns. Women reported higher levels of depression and more social support. Biroli et al. demonstrate in a survey study in Italy, UK, and the US that lockdowns also affected family life. They report that men took an increasing share of childcare, and especially grocery shopping. Women overall do more, and families with increased reallocation report greater tensions. Attanasi et al. show in a survey conducted in France that lockdowns may also affect gender-related potentially addictive behaviors. That is, women were more likely than men to report losing control of their usual diet and having increased smartphone usage, while no significant gender difference was detected for increased video game play. Furthermore, Michailidou focuses on differential treatment of men and women with respect to the (hypothetical) allocation of scarce medical resources among COVID-19 patients. In an online choice experiment with US participants, she finds that female and less healthy “patients” are treated preferentially, while people make no difference between more or less wealthy patients. Finally, in a review paper, Morgan et al. summarize how people of different genders are differentially affected by COVID-19 and why this is the case. The authors show that—while it is important to understand the different ways the groups are affected, discussing which group is most affected makes no sense.

## Summary and Future Directions

This Research Topic highlights the importance of a focus on gender when analyzing the outcomes of the COVID-19 pandemic. On the one hand, it turns out that in line with gender differences in economic preferences and personality traits, women and men perceive the crisis differently. This may affect their behavior in times of the crisis in a heterogeneous way in many domains (e.g., educational sector, labor market, households) that were subject to significant changes during the pandemic. On the other hand, the COVID-19 crisis has a different impact on women and men, which follows from their different situation in the labor market and the family. In this respect, the Research Topic demonstrates that gender differences—also beyond the male-female dichotomy—in the perception and impact of the crisis are ubiquitous. Norms and societal limitations affect these gender differences and their perception and effects. A better understanding of these mechanisms may help to tailor policies and information campaigns that address compliance, educational problems, political polarization, and well-being in lockdowns and beyond.

## Author Contributions

All authors listed have made a substantial, direct, and intellectual contribution to the work and approved it for publication.

## Conflict of Interest

The authors declare that the research was conducted in the absence of any commercial or financial relationships that could be construed as a potential conflict of interest.

## Publisher's Note

All claims expressed in this article are solely those of the authors and do not necessarily represent those of their affiliated organizations, or those of the publisher, the editors and the reviewers. Any product that may be evaluated in this article, or claim that may be made by its manufacturer, is not guaranteed or endorsed by the publisher.

## References

[B1] Albanesi S. Kim J. (2021). Effects of the covid-19 recession on the us labor market: occupation, family, and gender. J. Econ. Perspect. 35, 3–24. 10.1257/jep.35.3.3

[B2] Bierwiaczonek K. Kunst J. R. Pich O. (2020). Belief in COVID-19 conspiracy theories reduces social distancing over time. Appl. Psychol. 12, 1270–1285. 10.1111/aphw.12223 32864837

[B3] Branas-Garza P. Capraro V. Rascon-Ramirez E. (2018). Gender differences in altruism on mechanical turk: expectations and actual behaviour. Econ. Lett. 170:19–23. 10.1016/j.econlet.2018.05.022

[B4] Campos-Mercade P. Meier A. N. Schneider F. H. Wengström E. (2021). Prosociality predicts health behaviors during the COVID-19 pandemic. J. Public Econ. 195, 104367. 10.1016/j.jpubeco.2021.104367 PMC7842154 33531719

[B5] Capraro V. Barcelo H. (2020). The effect of messaging and gender on intentions to wear a face covering to slow down COVID-19 transmission. arXiv[Preprint].arXiv:2005.05467. 10.31234/osf.io/tg7vz PMC8013666 33821089

[B6] Charness G. Gneezy U. (2012). Strong evidence for gender differences in risk taking. J. Econ. Behav. Organ. 83, 50–58. 10.1016/j.jebo.2011.06.007

[B7] Croson R. Gneezy U. (2009). Gender differences in preferences. J. Econ. Lit. 47, 448–474. 10.1257/jel.47.2.448

[B8] Eckel C. C. Grossman P. J. (1998). Are women less selfish than men?: evidence from dictator experiments. Econ. J. 108, 726–735. 10.1111/1468-0297.00311

[B9] Galasso V. Pons V. Profeta P. Becher M. Brouard S. Foucault M. (2020). Gender differences in COVID-19 attitudes and behavior: panel evidence from eight countries. Proc. Natl. Acad. Sci. U.S.A. 117, 27285–27291. 10.1073/pnas.2012520117 33060298 PMC7959517

[B10] Hart P. S. Chinn S. Soroka S. (2020). Politicization and polarization in COVID-19 news coverage. Sci. Commun. 42, 679–697. 10.1177/1075547020950735 38602988 PMC7447862

[B11] Kerr J. Panagopoulos C. van der Linden S. (2021). Political polarization on COVID-19 pandemic response in the united states. Pers. Individ. Dif. 179, 110892. 10.1016/j.paid.2021.110892 PMC8631569 34866723

[B12] Keser C. Rau H. A. (2022). “Policy incentives and determinants of citizens' COVID-19 vaccination motives,” in University of Göttingen Working Paper in Economics, No. 434.

[B13] Mesch D. J. Brown M. S. Moore Z. I. Hayat A. D. (2011). Gender differences in charitable giving. Int. J. Nonprofit Volunt. Sector Market. 16, 342–355. 10.1002/nvsm.432

[B14] Meyers-Levy J. Loken B. (2015). Revisiting gender differences: what we know and what lies ahead. J. Consum. Psychol. 25, 129–149. 10.1016/j.jcps.2014.06.003

[B15] Müller S. Rau H. A. (2021). Economic preferences and compliance in the social stress test of the COVID-19 crisis. J. Public Econ. 194, 104322. 10.1016/j.jpubeco.2020.104322 PMC9186354 35702336

[B16] Niederle M. Vesterlund L. (2007). Do women shy away from competition? do men compete too much? Q. J. Econ. 122, 1067–1101. 10.1162/qjec.122.3.106733869490

[B17] Pummerer L. Böhm R. Lilleholt L. Winter K. Zettler I. Sassenberg K. (2022). Conspiracy theories and their societal effects during the COVID-19 pandemic. Soc. Psychol. Pers. Sci. 13, 49–59. 10.1177/19485506211000217

